# Cook County Medical Society

**Published:** 1855-04

**Authors:** 


*Cook County Medical Society—Variola.*

At the regular meeting of the Cook Co. Medical Society on the first Tuesday in February 1855, Dr. N. S Davis, chairman of the Committee on Epidemics, called the attention of members to the recent unusual prevalence of Small-Pox in the city. He said the disease had been developed with a rapidity and to an extent not easily explained on the supposition of contagion or simple communicability from one to another. Thus previous to the first of January there had been so few cases in the city that the subject had attracted no attention; only one case having come directly under the observation of the reporter. Between the sixth and the tenth of that month the disease sprung up as if by magic in almost every section of the city, and to some extent among all classes of the people. Twenty cases had come under his own care which had their beginning during that brief period. Six of these were unmodified by previous vaccination, the eruption was extensively confluent, and the constitutional symptoms very severe. The other 14 cases occurred in patients who had been previously vaccinated, and were modified so as to present almost every variety of Varioloid, from the slightest, with a mild premonitory fever and not more than a dozen pustules, to a degree of severity, closely resembling the milder cases of distinct Variola. From the 10th of January to the present time (Feb. 1st) only *three* new cases have come under his observation, and these were of the milder class of Varioloid Of the six unmodified cases two recovered without any untoward consequences, the other four presented symptoms of the most malignant or pernicious character from the beginning, and all proved fatal between the seventh and fourteenth days after the attack. One of these cases occurred in the person of a young man who was just recovering from a severe attack of Sub acute Gastritis.

The onset of the variolous fever was marked by a renewal of the gastric irritation, to such an extent that he vomited almost incessantly, accompanied by great distress and oppression at the epigastric region, a small and quick pulse, with extreme restlessness The eruption made its appearance at the end of the fourth day, but was accompanied by only a very moderate diminution of the gastric and constitutional symptoms. The eruption, too, was somewhat peculiar. Instead of presenting distinct pointed elevations in the skin, enlarging in circumference from day to day, and soon presenting a well marked indentation or umbilicated spot in the centre, they appeared mostly in large patches, the whole surface of which was *scarlet red*, and thickly studded over with minute vesicles, almost resembling clusters of herpes. One of these patches covered the entire face; and they were so numerous over the body and limbs, that after the third day of the eruption, scarcely the amount of six square inches of sound skin remained on the patient. The vesicles on these patches increased in size very slowly; only here and there one ever became umbilicated or distended with any fluid About the sixth day the scarlet redness began to fade, and the vesicles began to present the appearance of dried cuticle The swelling of the face was at no time as great as is often seen in mild cases of the disease. From the appearance of the eruption the pulse became a little slower, being about 110 per minute, and more full, but gaseous, or easily compressed There was much thirst and restlessness throughout the progress of the case About the seventh day of the eruption, the pulse became more frequent and weak, the mind dull and somewhat wandering, and dark purple spots appeared in many of the patches, both on the body and extremities. The patient, when aroused, also complained of a sense of extreme prostration or sinking in the epigastrium. From the appearance of these symptoms he sunk pretty rapidly, and died on the ninth day after the first appearance of the eruption. Two of the other fatal cases presented symptoms throughout strongly resembling the one just detailed. The premonitory fever was marked by extreme gastric irritation and distress; frequent and violent vomiting; only moderate pain in the head and back; the pulse from 110 to 120 per minute, rather small; the skin dry and hot; and in one of the cases frequent and bloody stools for two days. There was much thirst and great restlessness, but the most prominent point of complaint was the extreme distress in the epigastric region. In both these cases the eruption appeared on the fourth day, and presented the same general characteristics as described above In one of these cases the patches of *scarlet redness* appeared first on the hand and arms, and subsequently became almost continuous over the whole cutaneous surface. The appearance of these patches, their irregular development, their well defined deep red color, and the herpetic character of the vesicles, rendered them so peculiar that two or three of our most experienced physicians at first entertained doubts in regard to the nature of the disease. In both these cases death took place in what should have been the suppurative stage of the disease, and was preceded by the same purpuric spots and general typhoid symptoms as in the case first described.

The fourth fatal case presented no features differing from an ordinary casere of seve Variola until the completion of the suppurative stage, and gave every indication of recovery, when a copious diarrhœa commenced rather suddenly, ending in less than six hours, in a free hemorrhage from the intestines, and a speedy death. The fatal diarrhœa in this case occurred simultaneously with the inordinately severe snow storm of January.

It was remarked, that of the 20 cases alluded to as occurring between the sixth and the tenth of January, only one could be traced to any *known* exposure or contact with other cases. They were widely separated from each other in different sections of the city, and among different classes of people. The reporter said, if we add to these cases which had come under his own observations the numerous others which had occurred in the practice of other physicians, having their beginning during the same week. It will be clearly apparent that the city had been visited by a distinct Variolous Epidemic influence; and that too, of the same malignant character which, in the middle ages, so frequently decimated the population of European cities; and were it not that the great mass of the citizens are protected by vaccination, he had no doubt but we should now be in the midst of one of those loathesome pestilences, which make one shudder to contemplate. He stated that several instances had been reported during the last three weeks of attacks of varioloid in persons who had previously had the same disease.

The report was followed by a discussion, which was participated in by nearly all the members of the Society present at the meeting.

Dr. H. Smith said he had a female patient under his care with varioloid, who had had the same disease several years before, and had also the vaccine pustule in childhood.

Dr. Bloodgood doubted the full protective influence of vaccination, and expressed the opinion that a large majority of those who had been vaccinated were still susceptible in some degree to the variolous poison. He related several cases in which varioloid had followed recent vaccination.

Drs. Clarke and Miller expressed decided confidence in the full protective powers of vaccination when the virus is genuine. The latter mentioned a case of varioloid which he had met with during the past month, in the person of a female who had had distinct variola several years previous.

He also thought the vaccine virus ought to be renewed more frequently from the cow. To this Dr. C. G. Smith replied, that the experiments made by Mr. Ceely in England, and by several in Boston, with virus direct from the cow, seemed to show that such virus actually exerted no more protective influence than the ordinary matter in use.

Dr. N. S. Davis expressed strong confidence in the full protective power of the vaccine virus, and, as an illustration, mentioned the fact that in two families alone between twenty and thirty persons were fully exposed to the small-pox in its severest form during all of the second week in January. None of them had any other protection than that of a previous vaccination, and yet not one of the number took the disease even in its mildest form. In one of these families was a little child who had not been vaccinated until two days after she had been fully exposed. The vaccine virus took effect, and in due time produced the ordinary pustule on the arm. She, too, entirely escaped the variola.

NOTE.—Since the meeting of the society at which the above discussion took place, we have learned that about the same time that the small-pox appeared in this city in so unusual a degree, it appeared also in a very large proportion of the principal towns throughout the North-West: thus affording additional evidence of the existence of a true epidemic influence. We regret to learn, by the last Peninsular Journal, that at Ann Arbor it proved fatal to *three* members of the class attending the medical department of the Michigan University.

